# BOK controls ER proteostasis and physiological ER stress responses in neurons

**DOI:** 10.3389/fcell.2022.915065

**Published:** 2022-08-15

**Authors:** Franziska Walter, Beatrice D’Orsi, Anagha Jagannathan, Heiko Dussmann, Jochen H. M. Prehn

**Affiliations:** ^1^ Department of Physiology and Medical Physics, Royal College of Surgeons in Ireland University of Medicine and Health Sciences, Dublin, Ireland; ^2^ SFI FutureNeuro Research Centre, Royal College of Surgeons in Ireland University of Medicine and Health Sciences, Dublin, Ireland; ^3^ Institute of Neuroscience, Italian National Research Council, Pisa, Italy

**Keywords:** ER stress, Bcl-2 family, unfolded protein response, proteostasis, live-cell imaging, calcium signaling, ER stress reporters

## Abstract

The Bcl-2 family proteins BAK and BAX control the crucial step of pore formation in the mitochondrial outer membrane during intrinsic apoptosis. Bcl-2-related ovarian killer (BOK) is a Bcl-2 family protein with a high sequence similarity to BAK and BAX. However, intrinsic apoptosis can proceed in the absence of BOK. Unlike BAK and BAX, BOK is primarily located on the endoplasmic reticulum (ER) and Golgi membranes, suggesting a role for BOK in regulating ER homeostasis. In this study, we report that BOK is required for a full ER stress response. Employing previously characterized fluorescent protein-based ER stress reporter cell systems, we show that BOK-deficient cells have an attenuated response to ER stress in all three signaling branches of the unfolded protein response. Fluo-4-based confocal Ca^2+^ imaging revealed that disruption of ER proteostasis in BOK-deficient cells was not linked to altered ER Ca^2+^ levels. Fluorescence recovery after photobleaching (FRAP) experiments using GRP78/BiP-eGFP demonstrated that GRP78 motility was significantly lower in BOK-deficient cells. This implied that less intraluminal GRP78 was freely available and more of the ER chaperone bound to unfolded proteins. Collectively, these experiments suggest a new role for BOK in the protection of ER proteostasis and cellular responses to ER stress.

## Introduction

The endoplasmic reticulum (ER) provides an environment for the folding and post-translational modification of proteins in eukaryotic cells. Disruption of cellular homeostasis leads to an increase of unfolded proteins within the ER, causing ER stress. Consequently, a signaling network termed the unfolded protein response (UPR) is activated aimed at elevating the protein load and increasing the folding capacity of the ER (Hetz et al., 2020). In a first step, unfolded proteins are sequestered by the ER luminal chaperone GRP78, which dissociates from the transmembrane-signaling proteins IRE1, PERK, and ATF6, thereby activating them. Activated PERK phosphorylates translation initiation factor EIF2α, which leads to an attenuation of general translation and also translation initiation from the 5′UTR of transcription factor ATF4 (Harding et al., 1999). One of the transcriptional targets of ATF4 is CHOP, which has been associated with the pro-apoptotic cascade (Puthalakath et al., 2007; Han et al., 2013). Activated IRE1 catalyzes the splicing of XBP1 mRNA, resulting in the translation of transcription factor XBP1s and leading to enhanced expression of ER chaperones and folding factors (Calfon et al., 2002; Lee et al., 2003; Jonikas et al., 2009). The signaling outputs of the UPR either re-establish cellular homeostasis or result in inhibition of cell proliferation or induction of cell death (Tabas and Ron, 2011). ER stress-induced cell death occurs predominantly through the mitochondrial apoptosis pathway (Reimertz et al., 2003). The essential step in this pathway is the formation of pores in the outer mitochondrial membrane (MOM) by the pro-apoptotic Bcl-2 family members BAX and BAK, promoting the release of pro-apoptotic factors and caspase activation (Green and Llambi, 2015; Wei et al., 2001).

Bcl-2-related ovarian killer (BOK) shares a high sequence similarity with the pro-apoptotic members BAX and BAK (Hsu et al., 1997; Inohara et al., 1998). Several reports provide evidence for oligomerization of BOK and pore formation at the MOM, suggesting an effector function for BOK (Llambi et al., 2016; Fernandez-Marrero et al., 2017; Zheng et al., 2018). However, in a number of other studies, BOK deletion failed to be protective against apoptotic stimuli, implying additional roles for BOK independent of BAK/BAX-mediated MOM permeabilization (Carpio et al., 2015; D'Orsi et al., 2016; Echeverry et al., 2013). BOK is mainly localized at the ER and Golgi membranes (Echeverry et al., 2013), where it is bound to the inositol 1,4,5-trisphosphate receptors (IP_3_R) that regulate Ca^2+^ release from the ER (Schulman et al., 2013). This interaction has been shown to protect IP_3_Rs from proteolysis; however, it did not influence their Ca^2+^ mobilizing function. Furthermore, BOK stability has been demonstrated to be dependent on the interaction with IP_3_Rs, as unbound BOK is degraded rapidly by the proteasome (Schulman et al., 2016). The amount of free BOK appears to be tightly regulated in cells (Llambi et al., 2016; Schulman et al., 2016). Recently, BOK was reported to promote ER stress-induced apoptosis through calcium transfer at mitochondrial contact sites (Carpio et al., 2021).

BOKs localization at the ER membrane may suggest a function in ER proteostasis, ER stress signaling, or ER stress-induced cell death. BOK-deficient cells have been described as more sensitive to brefeldin A-induced ER stress (Echeverry et al., 2013). Furthermore, IRE1 and GRP78 expression were reported to be disrupted in *Bok* ^−/−^ mouse embryonic fibroblasts (MEF) (Echeverry et al., 2013). On the contrary, Carpio et al. (2021)found that loss of *Bok* protected cells from ER stress induced by thapsigargin and brefeldin A, caused increased eIF2α-phosphorylation but reduced transcription induction of ATF4 and CHOP (Carpio et al., 2015). Furthermore, PERK inhibition during tunicamycin-induced ER stress resulted in stabilization of BOK and cell death induction (Llambi et al., 2016).

In this study, we employed our previously characterized ER stress reporter cell lines in live-cell (HCS) imaging assays (Walter et al., 2015; Walter et al., 2018) to investigate how loss of BOK expression affected the UPR-signaling pathways. Furthermore, we utilized confocal Ca^2+^ imaging and fluorescence recovery after photobleaching (FRAP) assays to explore whether BOK affected the availability of Ca^2+^ or free GRP78 within the ER.

## Results

### *Bok*-deficient neurons have higher basal ER stress levels

**FIGURE 1:**
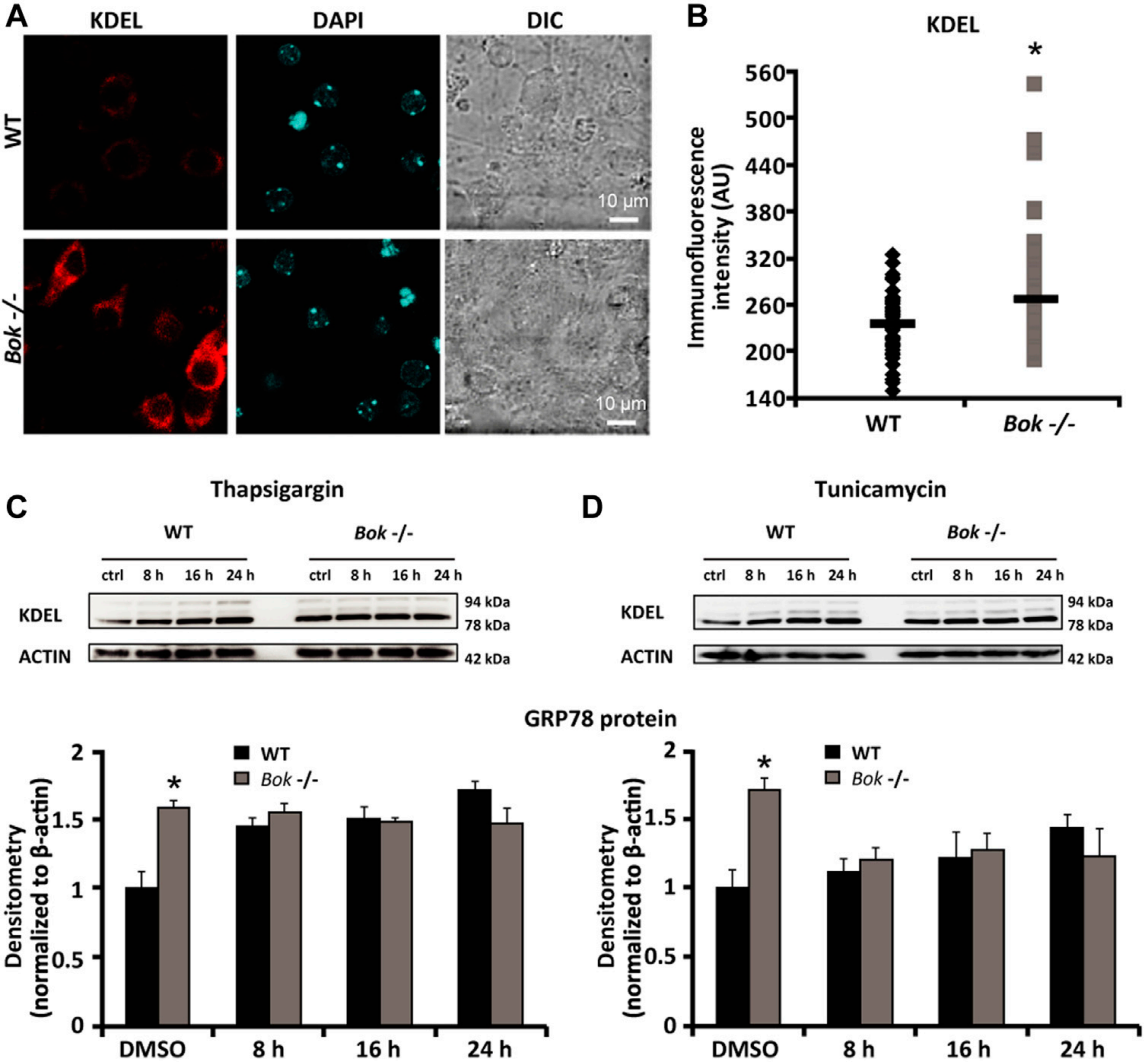
*Bok*-deficient neurons display higher basal ER stress. **(A,B)** Cortical neurons from WT and *Bok* ^*−/−*^ mice were stained for KDEL, an ER marker. Differential interference contrast (DIC), KDEL, and DAPI fluorescent images were captured from random fields using confocal fluorescence microscopy and chosen from a representative experiment [**(A)**, scale bar = 10 μm]. Analysis of basal KDEL levels **(B)** quantified as mean fluorescence intensity in WT (*n* = 54) and *Bok* ^*−/−*^ (*n* = 62) neurons is represented. A significant increase in the whole-cell KDEL fluorescence of the *Bok* ^*−/−*^ neurons was identified. * indicates statistical significance (*p* ≤ 0.05). *p*-value = 0.014 vs*.* WT cultures (Student’s t-test). **(C,D)** Western blot and densitometry analysis comparing the levels of GRP78 protein using a KDEL antibody in WT and *Bok* ^*−/−*^ cortical neurons either treated with thapsigargin [1 μM; **(C)**] or tunicamycin [3 μM; **(D)**] for the indicated times (8, 16, and 24 h) or exposed to the control (DMSO; 0.1% for 24 h). β-Actin was used as a loading control. Experiments were repeated at least three times with different preparations and similar results. Means ± SEM are shown. * indicates statistical significance (*p* ≤ 0.05). *p*-values are the following: *p* = 0.042 (DMSO *Bok* ^*−/−*^, thapsigargin) and *p* = 0.05 (DMSO *Bok* ^*−/−*^, tunicamycin) vs*.* WT cultures (Student’s t-test).

Having previously identified that BOK is highly expressed in neurons of the mouse brain and involved in controlling neuronal intracellular Ca^2+^ homeostasis (D'Orsi et al., 2016), we aimed to explore the potential role of BOK during ER proteostasis in primary cortical neuron cultures. We first evaluated the expression levels of the key ER stress markers GRP78 and GRP94 in cortical neurons derived from WT and *Bok*-deficient mice using immunocytochemistry and the KDEL antibody that recognizes a common Lys-Asp-Glu-Leu motif in these two proteins. Quantification of the whole-cell fluorescence intensity demonstrated a significant increase in the KDEL immunofluorescence in *Bok*-deficient neurons compared with their WT controls (Figures 1A,B). We next examined the effect of *Bok* ablation on ER stress responses induced by thapsigargin and tunicamycin, which inhibit the sarco/endoplasmic reticulum Ca^2+^-ATPase (SERCA) and N-linked glycosylation, respectively (Duksin and Mahoney, 1982; Lytton et al., 1991). Quantitative Western blotting analysis confirmed that loss of BOK protein increased GRP78 expression levels at basal (DMSO) conditions but not when neurons were exposed to thapsigargin- (Figure 1C) or tunicamycin- (Figure 1D) induced ER stress, suggesting that *Bok*-deficient primary neurons experienced a constant, basal level of ER stress.

### Loss of BOK expression affects all three branches of the ER stress response

**FIGURE 2:**
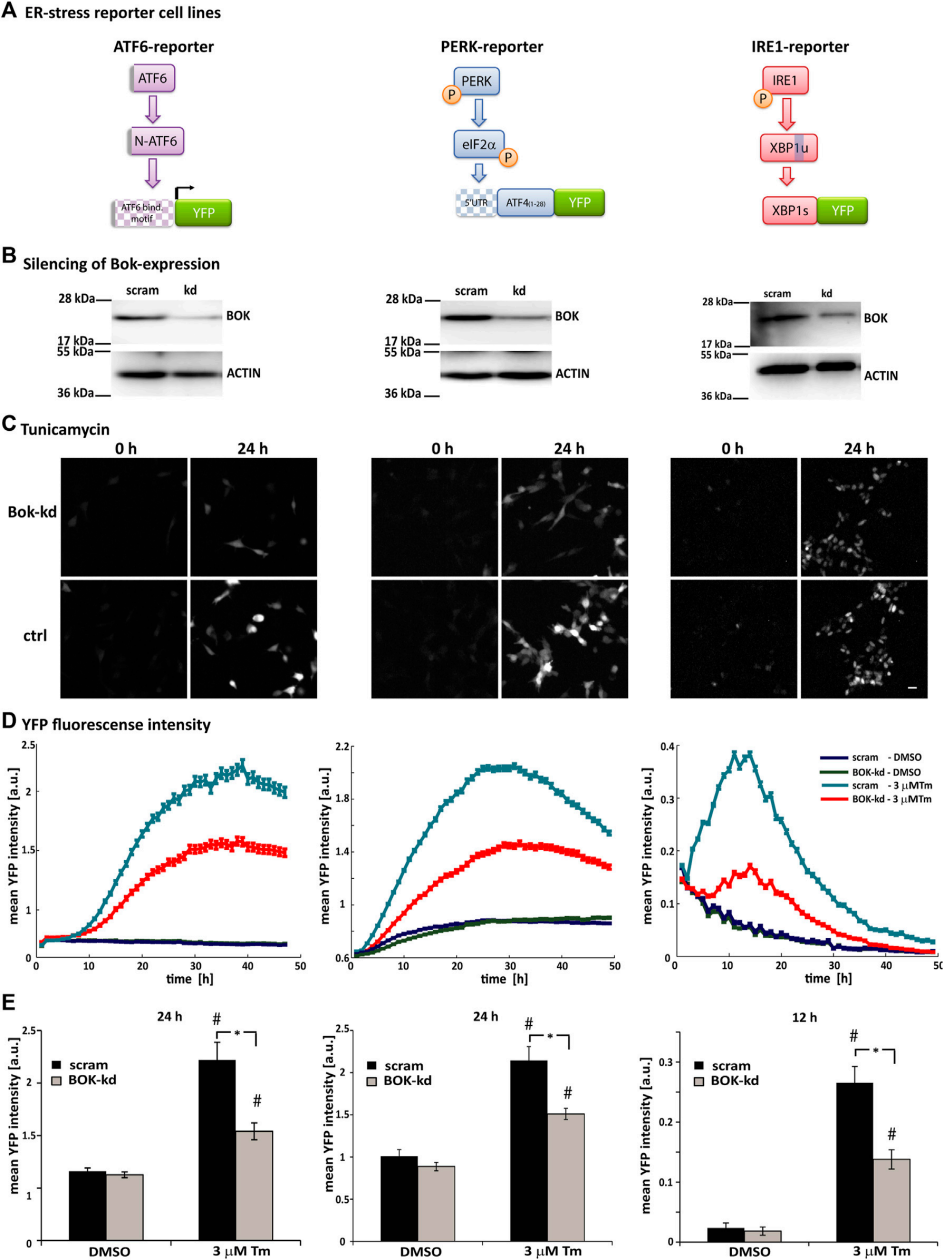
Loss of BOK expression affects all three branches of the ER stress response. **(A)** Schematic showing the principle of the ATF6-, PERK-, and IRE1-fluorescent reporter constructs. Under ER stress, cleaved N-terminal ATF6 binds to the 5xATF6-binding motif and initiates transcription of the YFP reporter. PERK phosphorylates translation initiation factor eIF2α, which initiates translation from the 5′UTR of the YFP reporter. Active IRE1α splices *Xbp1* mRNA, which removes a stop codon and enables expression of the XBP1-YFP reporter fusion protein. (**B)** SH-SY5Y cell lines stably expressing the ATF6-, PERK-, or IRE1-reporter were transduced with shRNA against BOK or scrambled control vector. Silencing of BOK protein expression was confirmed by Western blotting using the antibody against BOK. Actin served as a loading control; 72 h after transductions, cells were stained with Hoechst and PI and treated with 3 μM Tm or 0.1% DMSO. Images were taken at 1-h intervals starting immediately after treatment for 48 h using high-content time-lapse live-cell imaging. **(C)** Images taken in the YFP channel 0 and 24 h after treatment with 3 μM Tm of ATF6- (left panel), PERK- (middle panel), and IRE1-reporter cells (right panel) silenced for BOK expression and control cells (scale bar = 20 μm). **(D)** Mean YFP intensity over time of ATF6- (left panel), PERK- (middle panel), and IRE1-reporter cells (right panel) in response to 3 μM Tm or 0.1% DMSO in the BOK-kd and scrambled control group plotted over time. Error bars indicate SEM of n = 5 wells. **(E)** Mean YFP intensity 24 h (ATF6- and PERK-reporter) or 12 h (IRE1-reporter) after treatment with 3 μM Tm or 0.1% DMSO control. Error bars indicate SEM of *n* = 15 wells of three independent experiments*.* T-tests were performed comparing BOK-kd and scrambled control groups (* indicates *p* < 0.01) or DMSO- and tunicamycin-treated groups (# indicates *p* < 0.01).

To further investigate the role of BOK during ER stress, we used human SH-SY5Y neuroblastoma-derived ER stress reporter cell lines, which had been generated and extensively characterized in our laboratory (Walter et al., 2015; Nolan et al., 2016; Walter et al., 2018). These cell lines stably express fluorescent reporter constructs for each of the three branches of the UPR: 1) activation of ATF6 monitored through ATF6-dependent expression of YFP, 2) translation initiated from the 5′UTR region of ATF4 following PERK-dependent phosphorylation of eIF2α, and 3) splicing of XBP1 by IRE1 (Figure 2A).

In order to silence the expression of BOK, the reporter cell lines were transduced with lentiviral-delivered shRNA against *BOK*. Reduced levels of BOK were confirmed by Western blotting (Figure 2B). After transduction, the reporter cells were treated with tunicamycin and the expression of the fluorescent reporters was monitored using HCS imaging (Figures 2C–E).

In this system, we did not observe a difference in basal fluorescence intensity between the DMSO-treated BOK-kd and control cells. However, we found that in all three reporter cell lines the mean YFP fluorescence intensity was significantly lower in cells where BOK expression had been silenced compared with control cells. While the time of onset of fluorescence was similar in BOK-kd and control cells (Figure 2D), the mean fluorescence intensity was significantly lower 24 h after treatment in ATF6- and PERK-reporter cells and 12 h after tunicamycin treatment in IRE1-reporter cells (Figure 2E). This suggested that while BOK-deficient cells responded to ER stress by initiating the UPR at the same time as control cells, neither the ATF6-, nor the PERK- or IRE1-pathway were fully activated in cells without BOK expression. Taken together our experiments show that loss of BOK expression affected all three branches of the ER stress response in SH-SY5Y-derived cells.

### Reduced ER stress signaling is a general response in BOK-deficient cells that can be rescued through overexpression of BOK

To investigate whether reduced activation of the UPR was a general response to ER stress in BOK*-*deficient cells, we also treated the reporter cell lines transduced with the BOK-kd construct with thapsigargin and brefeldin A, the latter inducing ER stress by inhibiting protein transport from the ER to the Golgi apparatus (Supplementary Figures S1A–F). HCS imaging showed that BOK-deficient cells had lower levels of fluorescence intensity in response to the ER stressors compared with control cells. This was the case for ATF6-, as well as for PERK- and IRE1-reporter cells. Analysis of three independent experiments revealed that the YFP mean fluorescence intensity was significantly lower 24 h after treatment with thapsigargin or brefeldin A in the ATF6- and PERK-reporter cells and 12 h after ER stress induction in the IRE1-reporter cell line. Similar to what we observed in tunicamycin-treated cells, the fluorescence intensity started to increase at the same time in both BOK-kd and control cells; however, the maximum fluorescence intensity reached in the BOK-kd cells remained at all times lower than in the controls. These results indicated that the attenuated ER stress response observed in BOK-deficient cells also occurred when other ER stress-inducing agents were used. Once again, we did not observe a difference in basal fluorescence intensity between the DMSO-treated BOK-kd and controls in SH-SY5Y neuroblastoma cells.

**FIGURE 3:**
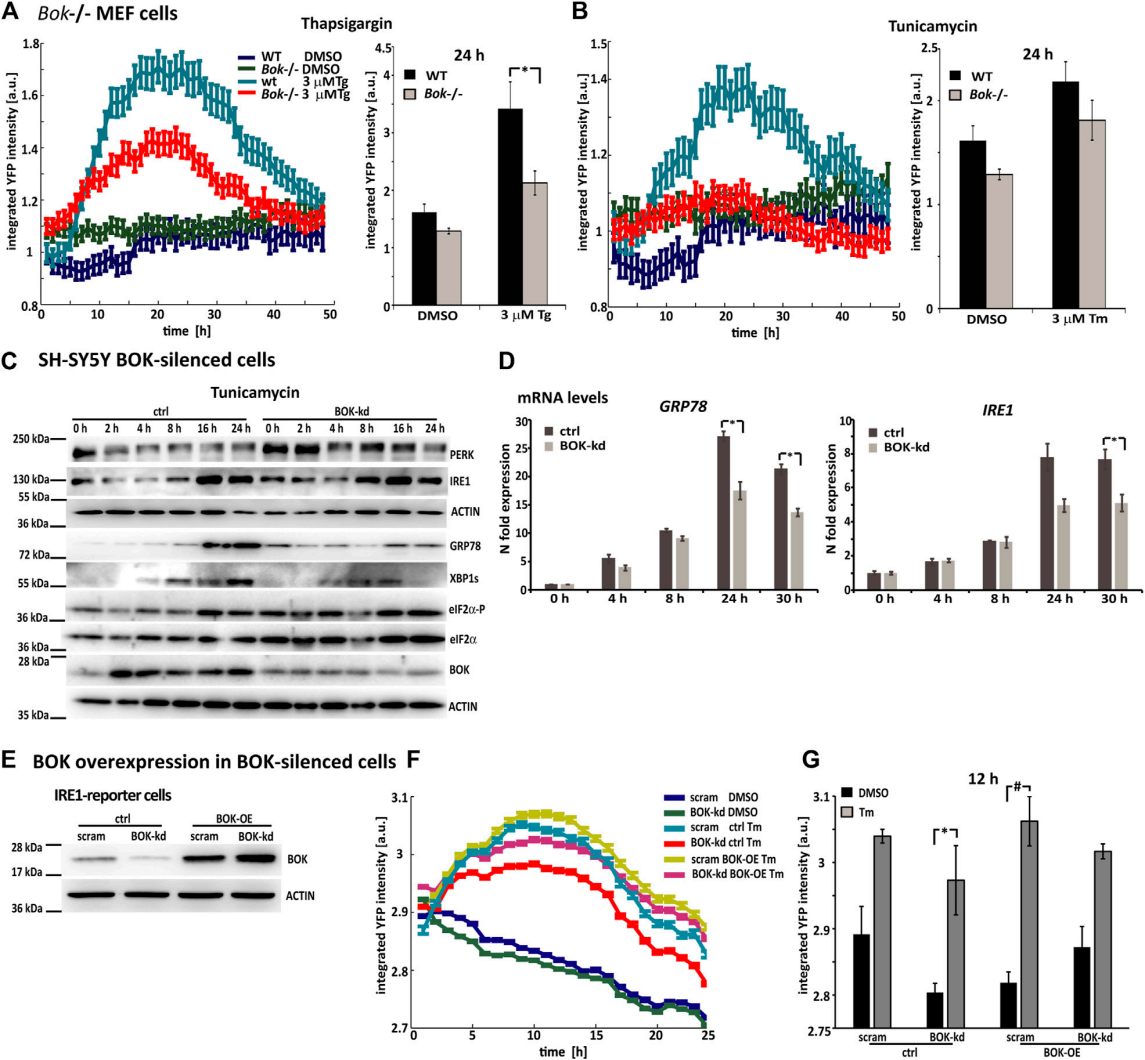
Reduced ER stress signaling is a general response in BOK-deficient cells, which can be rescued through overexpression of BOK. **(A)** Mouse embryonic fibroblasts from WT or *Bok* ^−/−^ knock-out mice were transduced with viral particles containing the PERK-reporter construct; 72 h after transductions, cells were stained with Hoechst and PI and treated with 3 μM Tg and **(B)** 3 μM Tm or 0.1% DMSO. Images were taken at 1-h intervals starting immediately after treatment for 48 h using high-content time-lapse live-cell imaging. The mean YFP intensity over time of the PERK-reporter in the *Bok* ^−/−^ and WT cells was plotted. Error bars indicate SEM of at least *n* = 2 wells. Bar graphs show mean YFP intensity 24 h after treatment with 3 μM Tg or 3 μM Tm or 0.1% DMSO control. Error bars indicate SEM of at least *n* = 7 wells of three independent experiments*.* Student’s t-tests were performed comparing *Bok* ^−/−^ and WT. * indicates *p* < 0.01. **(C)** SH-SY5Y cells stably expressing shRNA against *BOK* or control vector were treated with 3 μΜ Tm and harvested at the times indicated. Expression of UPR proteins was analyzed by Western blotting using antibodies against PERK, IRE1, KDEL, spliced XBP1s, eIF2α-P, eIF2α, and BOK. Actin served as a loading control. The experiment was repeated with similar results. **(D)** Real-time qPCR analysis of *GRP78*- and *IRE1*-mRNA levels in SH-SY5Y BOK-silenced- and control cells treated with 3 μΜ Tm. Results were normalized to β-actin levels and expressed relative to 0 h control- or BOK-kd cells, respectively (mean of *n* = 3 wells, error bars indicate SEM, and Student’s t-tests were performed comparing control and BOK-kd; * indicates *p* < 0.05). **(E)** IRE1-reporter cells stably expressing shRNA against *BOK* or control vector were transfected with BOK overexpression construct hBok (CDS)-pcDNA or empty pcDNA3.1 plasmid. Expression of BOK was analyzed by Western blotting using antibodies against BOK. Actin served as a loading control. The experiment was repeated with similar results. **(F)** After 24 h of transfection, cells were stained with Hoechst and PI and treated with 3 μM Tm or 0.1% DMSO. Images were taken at 1-h intervals starting immediately after treatment for 25 h using high-content time-lapse live-cell imaging. The mean YFP intensity over time of the IRE1-reporter in the BOK-kd and control cells was plotted. Error bars indicate SEM of at least *n* = 3 wells. **(G)** Bar graphs show mean YFP intensity 12 h after treatment with 3 μM Tm or 0.1% DMSO control. Error bars indicate SEM of at least *n* = 3 wells*.* Student’s t-tests were performed comparing Tm- and DMSO-treated cells. * indicates *p* < 0.05; # indicates *p* = 0.05.

As BOK expression was significantly reduced, but not entirely deleted, in the SH-SY5Y reporter cells, we also conducted experiments in *Bok* ^*−/−*^ MEF cells that were transduced with lentiviral particles carrying the PERK-reporter construct (Figures 3A,B). After transduction, expression levels of the fluorescent PERK-reporter in response to thapsigargin or tunicamycin were monitored using HCS imaging. Once more, we observed that YFP fluorescence intensity in *Bok* ^*−/−*^ MEF cells was lower compared with WT cells. PERK-reporter expression was significantly lower 24 h after thapsigargin treatment in *Bok* ^−/−^ compared with WT control cells, suggesting that the reduced response to ER stress occurred in BOK-deficient cells independently of the method used to silence BOK expression.

We also assessed expression levels of UPR-signaling proteins in BOK-deficient SH-SY5Y parental cells using Western blotting and qPCR. To this end, we generated an SH-SY5Y cell line, which stably expressed shRNA against *BOK*. The *BOK*-silenced cells and control cells were treated with tunicamycin and harvested at different time points, as indicated (Figure 3C). Western blotting showed that levels of UPR-signaling proteins IRE1, GRP78, and XBP1s were diminished in the BOK-deficient cells compared with their controls 16 and 24 h after ER stress induction. This is further supported by *GRP78* and *IRE1* mRNA levels being lower in the BOK-kd compared with control cells (Figure 3D).

Additionally, we analyzed levels of UPR-signaling proteins in MEF *Bok* ^*−/−*^ cells in response to tunicamycin treatment (Supplementary Figure S2A). Similar to our observation in cortical neurons, we found that levels of GRP78 were elevated at 0 h in the *Bok* ^−/−^ cells. Increase in GRP78, IRE1, and XBP1s levels in response to tunicamycin was lower in the *Bok* ^−/−^ cell compared with WT. We also found reduced *Grp78* mRNA levels in the *Bok* ^−/−^ MEF cells 24 h after Tm treatment (Supplementary Figure S2B).

Finally, to reconstitute BOK function, SH-SY5Y-derived ER stress reporter cell lines stably expressing shRNA against *BOK* were transfected with BOK overexpression constructs. BOK protein levels were assessed by Western blotting (Figure 3E). The reporter cells were treated with tunicamycin, and YFP fluorescence intensity was imaged for 24 h. We found that mean fluorescence intensity levels in BOK-kd cells that were transfected with the BOK overexpression construct were similar to those in scram control cells transfected with empty control vector (Figures 3F,G). However, in BOK-kd cells transfected with control vector, the fluorescence intensity was lower, indicating a lower ER stress response. This suggested that the reduced ER stress response was due to missing BOK protein levels, as re-introduction of BOK alleviated the effect.

As reduced ER stress signaling appeared to be a general response in cells without physiological BOK levels, we wanted to investigate whether this failure to fully activate the ER stress response affected cell death. To this end, SH-SH5Y cells stably expressing shRNA against BOK or control vector and *Bok* ^−/−^ MEF cells and WT controls were stained with Hoechst and PI or treated with ER stress inducers tunicamycin or thapsigargin and fluorescence was monitored for 45 h using HCS imaging (Supplementary Figure S3).

In SH-SH5Y control cells, levels of PI-positive cells were low in the beginning and increased sharply 30 h after treatment. We found that cell death levels in the SH-SY5Y BOK-kd cells were higher than those in the control at the outset; it increased early over the course of the experiment, but then plateaued. (Supplementary Figures S3A,B). In non-transformed *Bok* ^−/−^ MEFs cells, the percentage of PI-positive cells was consistently higher than in the WT cells throughout the exposure to the ER stressors (Supplementary Figures S3C,D). Taken together, our experiments suggest that BOK-deficient cells are more vulnerable to ER stress. However, cell death responses are dependent on the system and time points under study.

### BOK deficiency does not result in ER calcium dysregulation

**FIGURE 4:**
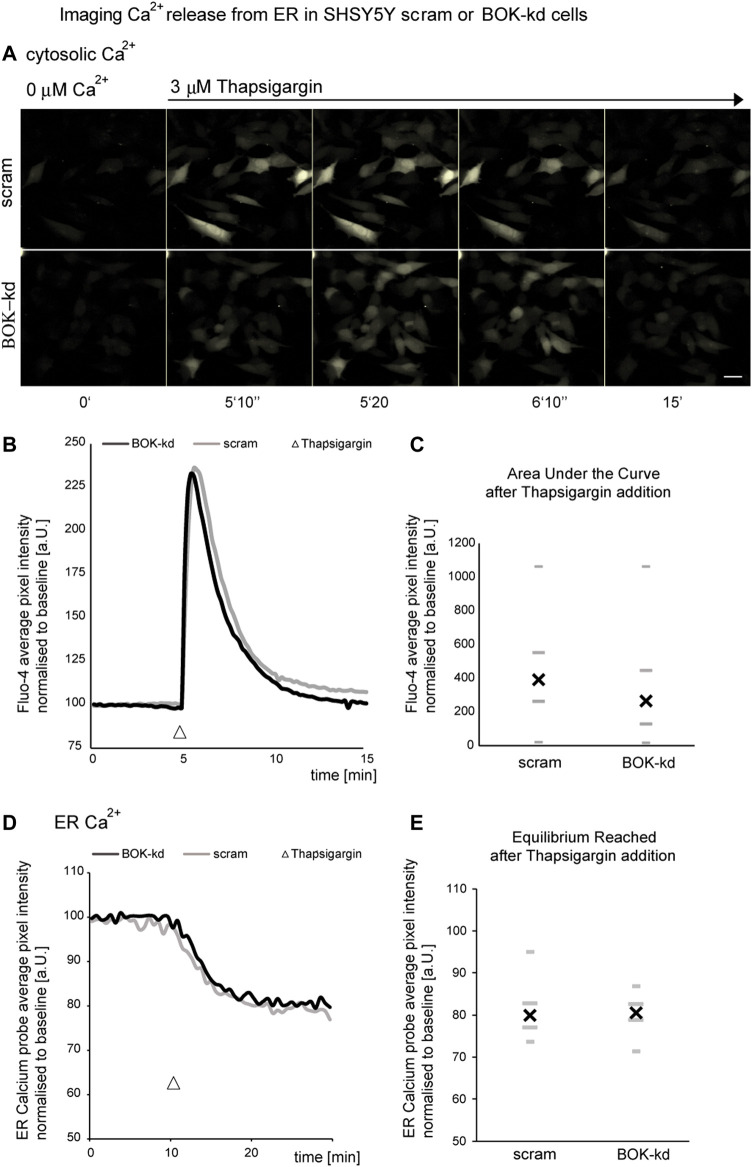
BOK deficiency does not result in ER calcium dysregulation. SHSY5Y cells stably expressing shRNA against *BOK* or scram control stained with Fluo-4 were imaged in Krebs buffer without extracellular calcium every 10 s. **(A,B)** Treatment with 3 μM thapsigargin caused a rapid transient spike in fluorescence intensity. **(A)** Representative images of one out of four experiments are shown (scale bar 20 μm**)**. **(B)** Average kinetics for all experiments, (*n* = 79 cells BOK-kd or ctrl analyzed for all experiments). **(C)** There was no difference in the median overall area under the curve above between scram control and BOK-kd cells (* indicates *p* = 0.3942; Mann–Whitney U-test). SHSY5Y cells stably expressing shRNA against *BOK* or scram control, transfected with the ERD1CPV vector, were imaged in Krebs buffer without extracellular calcium every 30 s. **(D,E)** Treatment with 3 μM thapsigargin caused a drop in the FRET ratio (FRET/CFP normalized to baseline as measured at the start of the experiment) indicating the loss of Ca^2+^ from ER lumen **(D)**. The normalized FRET/CFP drops to a similar value independent of BOK expression **(E)**, *n* = 39 (scram) and *n* = 48 (BOK-kd) SHSY5Y cells from four experiments for each cell type, median (X), and quartiles **(-**,^**_**^; Mann–Whitney U-test, *p* = 0.5).

Previous studies have shown that BOK is constitutively bound to the inositol 1,4,5-trisphosphate receptors (IP_3_Rs) (Schulman et al., 2013). IP_3_Rs form channels in the ER membrane and control the release of Ca^2+^ from the ER. Because ER Ca^2+^ is required for protein folding, a reduction in ER Ca^2+^ levels may induce continuous ER stress that could cause a defective and reduced sensitivity to further ER stressors. Thus, we next investigated whether BOK deficiency caused alterations in ER Ca^2+^ levels by quantifying Ca^2+^ release from the ER in cells treated acutely with the ER Ca^2+^ ATPase inhibitor thapsigargin. BOK-deficient SH-SY5Y cells were loaded with 5 µM Fluo-4-AM, placed in a buffer solution without Ca^2+^ and exposed to 3 µM thapsigargin. We found that thapsigargin induced a similar release of ER Ca^2+^ characterized by a rapid transient spike in intracellular Ca^2+^, in both SH-SY5Y cells stably expressing shRNA against BOK and in scrambled control cells (Figures 4A–C). We found no significant difference in the area under the curve between BOK-kd and control cells, suggesting that BOK is not required for release of Ca^2+^ from the ER (Figure 4C).

Next we transfected BOK-kd and control cells with an ER-calcium probe [ERD1CPV, (Palmer et al., 2006)] to measure calcium levels within the ER in response to 3 µM thapsigargin (Figures 4D,E). We observed no difference in the rate of calcium release from the ER following thapsigargin treatment. Taken together our experiments suggest the BOK deficiency does not result in altered calcium release from the ER in the SH-SY5Y-derived BOK-kd cells.

### GRP78 mobility is reduced in mouse embryonic fibroblast *Bok* ^−/−^ cells

**FIGURE 5:**
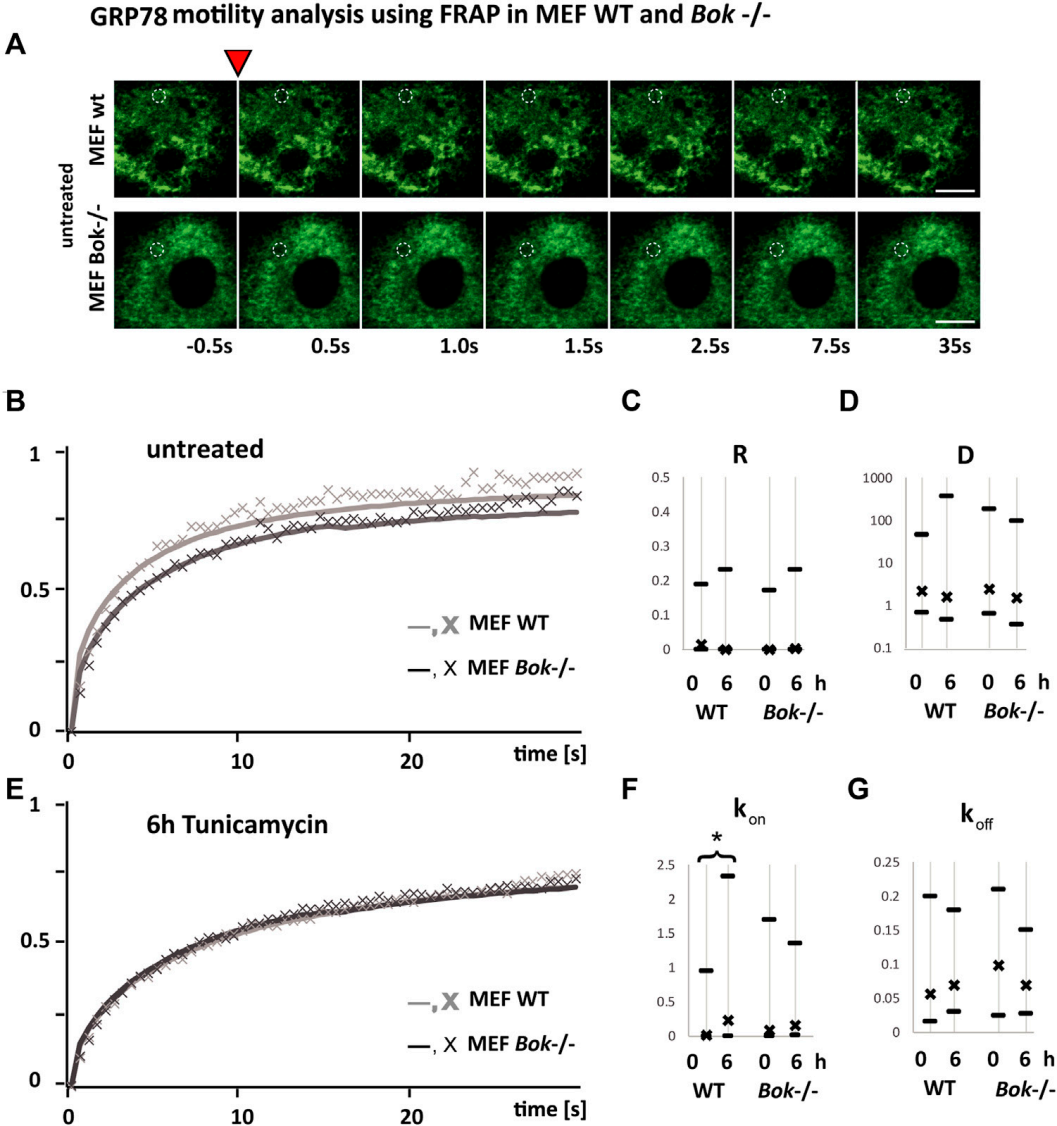
GRP78 mobility is reduced in MEF *Bok* ^−/−^ cells. FRAP recovery kinetics of a 1.7 μm bleached spot in the ER area of MEF WT (light gray line and symbol) compared with MEF *Bok* ^−/−^ (dark gray line and symbol) cells transfected with BiP-eGFP. **(A)** Examples from FRAP imaging sequences comparing MEF WT and MEF *Bok* ^−/−^ BiP-eGFP-expressing cells. The inverted red triangle indicates the photobleaching step, times of imaging after the bleach step are indicated below, and the bleached area is labeled with a dashed white circle (scale bar = 10 μm). **(B,E)** Lines represent the mean of all fitted curves, and the X symbol represents the mean of the measured kinetics normalized to the maximum difference of baseline and bleached and corrected for bleaching during measurements. The curves show kinetics before **(B)** and after 6 h **(E)** of treatment with 3 μΜ tunicamycin. Comparison of the recovery kinetics median and the 25 and 75% quartiles using the four fitting parameters, the immobile fraction (R), the diffusion **(D)**, the binding (k_on_), and the dissociation (k_off_) of BiP-eGFP **(C,D,F,G)**. The binding (k_on_) is significantly increased after 6 h in WT but not in *Bok* ^−/−^. A minimum of 94 FRAP curves from three independent experiments were analyzed per group (WT, untreated *n* = 112; WT, 6 h Tm *n* = 94; *Bok* ^*−/−*^, untreated *n* = 98; *Bok* ^*−/−*^, 6 h Tm *n* = 99); * indicates a significant difference using the Kruskal–Wallis and Dunn’s test; # indicates two-tailed t-test, non-homogenous variances, *p* = 0.01.

We next hypothesized that the dampened ER stress response in *Bok*-deficient cells may be due to an enhanced occupancy of GRP78 with unfolded proteins that could result in a reduced availability for ER stress signaling when cells are further exposed to stress. Hence, we performed FRAP assays to measure the motility of GRP78 (Lai et al., 2010) (Figure 5). The rate at which fluorescence is recovered after photobleaching reflects the motility of GRP78 with fluorescence recovery being faster if more GRP78 within the ER is unbound. Reversibly, we expect a slower recovery if most of the available GRP78 is occupied by unfolded proteins. MEF cells derived from *Bok* ^−/−^ and WT mice were transiently transfected with BiP-eGFP and imaged before and 6 h following tunicamycin treatment. A small circular area of the ER, where the probe was expressed, was bleached, and subsequently, images were taken at 0.5 s intervals to monitor the recovery of fluorescence in the bleached area (Figure 5A). We observed slower recovery kinetics in untreated *Bok* ^−/−^ cells compared with WT cells, caused by an initially lower mobility of BiP-GFP in the *Bok* ^*−/−*^ cells (Figure 5B). After 6 h of tunicamycin treatment which induces binding of GRP78 to unfolded proteins, we observed that FRAP kinetics in the WT and *Bok* ^−/−^ cells were similar, suggesting that as a result of ER stress occupancy of GRP78 in the WT cells reached similar levels to that in the *Bok*-deficient cells (Figure 5E).

The recovery kinetics were fitted using a function for diffusion and binding kinetics in a circular spot (Sprague et al., Equation 6 employed in FRAP analyzer software by A. Halavatyi) (Sprague et al., 2004). This allowed a comparison of recovery kinetics based on the non-mobile fraction R, the diffusion D, binding constant k_on_, and dissociation constant k_off_ of BiP-eGFP from any binding partner.

We observed that there was no significant difference in the non-mobile fraction (Figure 5C) or the diffusion constant (Figure 5D) of BiP-eGFP between WT and *Bok* ^−/−^ MEF cells, either before or after tunicamycin treatment. The dissociation of BiP-eGFP binding into complexes as fitted with k_off_ was also not affected significantly in any of the conditions. In contrast, we detected that tunicamycin induced a significant increase in the binding constant k_on_ (Figure 5F) only in WT cells, but not in *Bok* ^−/−^ cells, suggesting that binding of GRP78 to target proteins is reduced in the *Bok* ^*−/−*^ cells.

Collectively, these experiments indicated that MEF cells without BOK function have a reduced mobility of GRP78 as reflected by the slower recovery shown in Figure 5B. These cells also show a reduced ER stress-signaling response (Figures 3A,B; Supplementary Figure S2A). The reduced mobility and UPR response observed in BOK-deficient MEF cells may be caused by increased occupancy of GRP78 with unfolded proteins and consequently result in attenuated UPR signaling.

## Discussion

In this study, we focused on elucidating the role of BOK during ER stress signaling through employing a systematic, single-cell reporter imaging approach, which we furthermore validated with bulk cell analysis. While previous studies focused on experiments and interpretation of single branches of the ER stress response, our approach allowed us to investigate all three branches of ER stress signaling.

We show that BOK is necessary to induce full UPR signaling in response to ER stress, and that in particular non-transformed BOK-deficient cells, such as neurons, may experience constant levels of ER stress. Our finding of a global attenuation of all three ER stress responses in BOK-deficient cells then let us to conduct detailed FRAP experiments, demonstrating that BOK is required for the regulation of ER resident chaperone BiP/GRP78 binding to misfolded proteins in the ER.

As BOK is a member of the Bcl-2 family with high sequence similarity to cell death effector proteins BAX and BAK, many studies have focused on elucidating the role of BOK during apoptosis. However, several recent reports proposed that BOK has functions beyond cell death signaling and the control of apoptosis (D'Orsi et al., 2016; Naim and Kaufmann, 2020). Because BOK is highly expressed in neurons (D'Orsi et al., 2016), we initially investigated ER stress signaling in cortical neurons. Using immunohistochemistry and quantitative Western blotting, we observed that the ER resident chaperone GRP78 was highly expressed under control conditions in *Bok*-deficient neurons. Increased GRP78 levels are a known hallmark of ER stress (Lee, 2005). Next, we found that additional exposure to ER stress failed to increase GRP78 expression in *Bok*-deficient neurons, which lead us to hypothesize that loss of BOK also disrupted UPR signaling and resulted in a failure to mount an ER stress response in these cells. To further investigate, neuroblastoma (SH-SY5Y)-derived ER stress reporter cell lines were employed and showed that BOK ablation dampened all three strands of UPR signaling. Notably, reconstitution of BOK rescued this phenotype. However, BOK did not alter the onset of fluorescence intensity increase in any of the reporter cell lines, suggesting that BOK may be necessary for the full volume, but not onset of the UPR. IRE1 and GRP78 expressions were previously reported to be disrupted in *Bok* ^−/−^ knockout mouse embryonic fibroblasts (MEF) (Echeverry et al., 2013). While these reports have shown that loss of BOK affects a specific branch of UPR signaling, such as IRE1- (Echeverry et al., 2013) or PERK-eIF2α-ATF4-signaling (Carpio et al., 2015), our data indicate that BOK deficiency influences indeed all three UPR-signaling branches in the neuroblastoma-derived cell lines.

Of note, we did not observe increased levels of basal ER stress in the BOK-silenced neuroblastoma cells. For instance, the fluorescence intensity in vehicle-treated BOK-deficient and control neuroblastoma cells remained similar throughout the experiments. It is possible that transformed neural cells have an increased capacity to withstand defects in ER proteostasis compared with non-transformed cells and/or post-mitotic neurons. Thus, the defect caused by loss of BOK only becomes apparent in the reporter cell lines when challenged with ER stress. Interestingly, increased occupancy of GRP78 in BOK-deficient MEFs as seen in the FRAP experiments also suggested that there were basal levels of ER stress present in this system.

As the role of BOK in ER stress-induced apoptosis remains controversial, we employed our live cell imaging set-up to investigate cell death in the SH-SY5Y BOK-kd cell line and the *Bok* ^−/−^ MEFs. We found that cell death levels in the SH-SY5Y BOK-kd cells are higher initially and increased early during the course of the experiment, but then plateaued. In contrast, we observed a sharp increase in cell death in SH-SY5Y control cells in response to ER stress, as we have described previously (Walter et al., 2015; Walter et al., 2018). While the BOK-kd cells appear to be constantly stressed there is no clear onset of cell death in response to ER stressors in these cells. As the BOK-deficient cells fail to mount an ER stress response, they might also “‘escape” ER stress-induced apoptosis mechanisms, which are activated after prolonged exposure*.*

Similarly, we found that *Bok* ^−/−^ MEF cells had higher cell death levels in response to ER stress throughout the experiment. The higher levels of cell death already at the beginning of the experiment would suggest that BOK-deficient cells experience constant stress and are thus more vulnerable to subsequent ER stressors. Furthermore, our experiments demonstrate that the exact response to ER stress varies depending on the cell type—primary or transformed cells, and the exposure time. This is in line with a study by Fernandez-Marrero et al. (2016) whose experiments did not indicate a pro-apoptotic role of BOK downstream of ER stress.

Our reporter experiments indicated that BOK impinges on ER stress responses upstream of IRE1-, PERK-, or ATF6- signaling. Because BOK has been shown to bind to IP_3_Rs in the ER membrane (Schulman et al., 2013; Schulman et al., 2016), we next elucidated whether BOK controlled ER Ca^2+^ levels required for protein folding in the ER. However, thapsigargin treatment in BOK-silenced SH-SY5Y cells displayed the same transient increase in intracellular Ca^2+^ as control cells, indicating that loss of BOK did not affect IP_3_Rs-mediated Ca^2+^ release. Schulman et al. made a similar observation in *Bok* ^−/−^ MEFs exposed to lysophosphatidic acid (LPA), which is able to stimulate IP_3_Rs formation (Schulman et al., 2013; Schulman et al., 2019). We additionally measured ER calcium levels, but found no difference in the rate of Ca^2+^ release from the ER following treatment with thapsigargin, which further supports our point that BOK is not involved in ER-Ca regulation. Our findings are also in line with experiments by Carpio et al. (2021) who measured cytosolic Ca^2+^ levels after thapsigargin treatment in WT and *Bok* ^−/−^ MEF cells.

**FIGURE 6:**
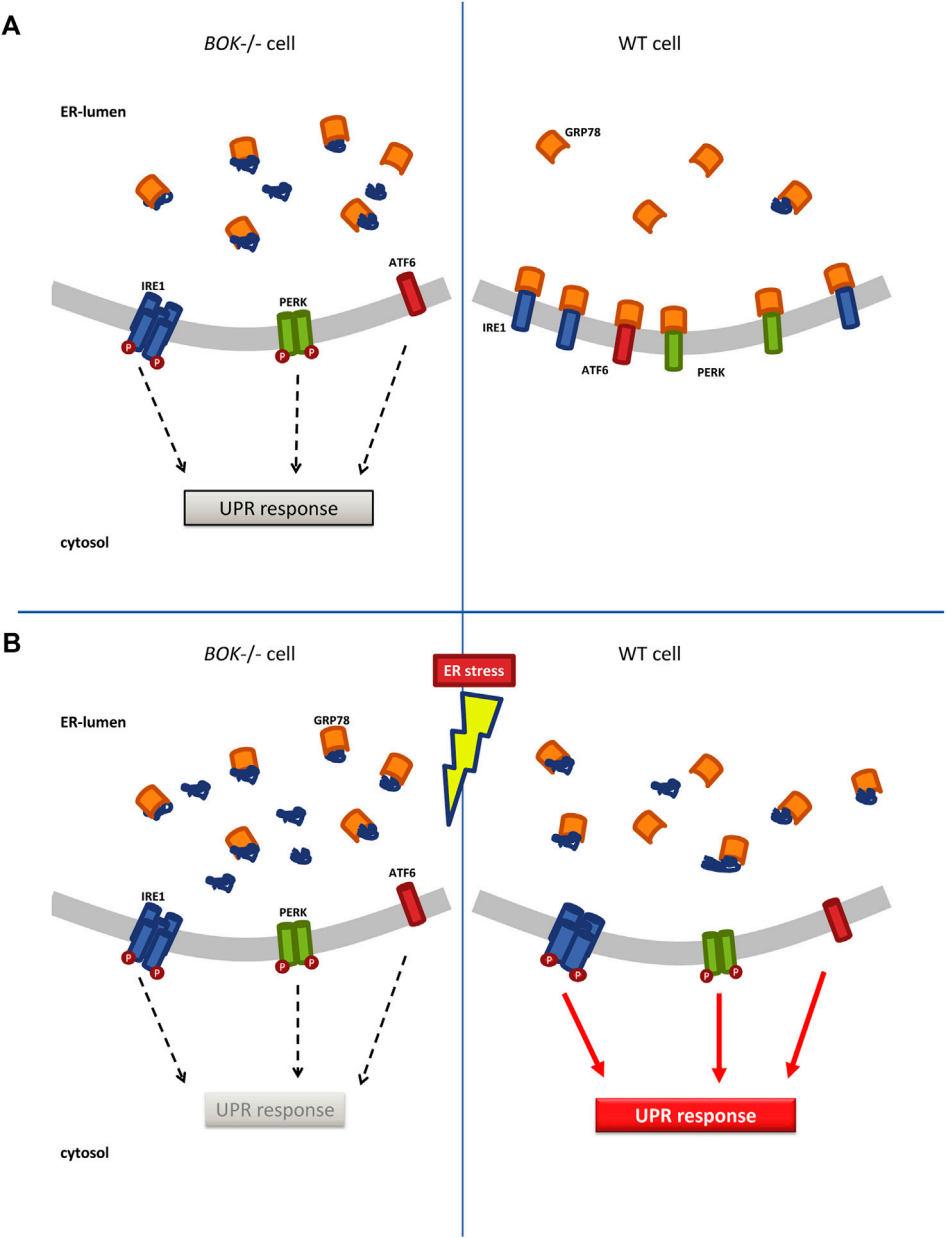
ER stress response in *Bok* ^−/−^ and WT cells. **(A)** *Bok* ^−/−^ cells experience chronic low levels of ER stress marked by lower motility of ER chaperone GRP78 indicating that GRP78 is bound to unfolded proteins. This allows for low-level oligomerization and activation of the membrane-signaling proteins IRE1, PERK, and ATF6, which initiate the UPR response. In contrast, in WT cells, free GRP78 is available in the ER and can bind to IRE1, PERK, and ATF6 keeping them in an inactive, monomeric state. **(B)** When *Bok* ^−/−^ cells are exposed to additional stress, they fail to further increase their ER stress signaling and cannot mount a full UPR response. In WT cells, exposure to ER stress increases the amounts of unfolded proteins in the ER, which leads to dissociation of GRP78 from IRE1, PERK, and ATF6 fully activating the UPR.

Under ER stress, GRP78 sequesters unfolded proteins as a first step of the UPR, a process key to all three branches of the UPR. We therefore investigated the availability of free GRP78 within the ER employing a FRAP assay (Lai et al., 2010). The motility of GRP78 is an indicator for the amount of its unbound level. A significant increase in binding of the BiP-eGFP probe to target proteins in response to tunicamycin was observed in WT cells but not in *Bok* ^−/−^ MEF cells. We hypothesize that in *Bok* ^*−/−*^ cells GRP78 is occupied by unfolded proteins even before induction of ER stress with tunicamycin, which explains the delayed FRAP recovery in the *Bok* ^−/−^ cells. The enhanced occupancy of GRP78 with unfolded proteins could result in a subsequent failure to initiate the UPR when BOK-deficient cells are exposed to additional stress. This would in turn lead to attenuated UPR signaling, which we observed in the *Bok* ^−/−^ MEF cells as well as in the SH-SY5Y derived ER stress reporter cell lines (Figure 6).

Collectively, our data suggest a dynamic regulation of intracellular GRP78 levels, their availability for protein folding in the ER and the activity or expression levels of BOK. We here report a novel non-apoptotic function for BOK. We show that BOK is required for ER proteostasis and the full induction of the ER stress response, with a loss of BOK resulting in attenuated signaling in all three branches of the UPR.

## Materials and methods

### Materials

Fetal bovine serum, horse serum, minimal essential medium (MEM), and B27-supplemented neurobasal medium came from Biosciences (Dun Laoghaire, Ireland). All other media and chemicals and dyes including tunicamycin, thapsigargin, and brefeldin A were obtained in analytical grade purity from Sigma-Aldrich, Ireland.

### Gene-targeted mice

The generation and genotyping of *Bok* ^*−/−*^ mice has previously been described (D'Orsi et al., 2016). *Bok* ^*−/−*^ mice generated by gene targeting using C57BL/6-derived Bruce4 embryonic stem (ES) cells (Ke et al., 2012) were bred as a homozygous knockout colony. As controls, wild type (WT) mice were used for all experiments. All mouse strains were backcrossed for >12 generations on an inbred C57BL/6 background. WT and gene-deficient mice were generated and maintained in-house in the RCSI Biological Research Facility.

The genotype of *Bok* ^*−/−*^ mice was confirmed by PCR. DNA was extracted from tail snips using the High Pure PCR Template Preparation Kit (Roche, Sussex, United Kingdom). Genotyping was performed using three specific primers as follows: 5′-CGG​GTT​TGA​ATG​GAA​GGG​TC-3′ (common forward primer), in combination with two reverse primers 5′-TGT​TCC​CAT​GGT​GCT​ACA​TCC-3′ and 5′-GAG​CTA​GCT​AGC​TAT​GTG​TG-3′ for *Bok*. All animal work was performed with ethics approval and under licenses granted by the Health Products Regulatory Authority (HPRA, Ireland) in accordance with European Communities Council Directive (86/609/EEC) and procedures reviewed and approved by the RCSI Research Ethics Committee.

### Preparation of mouse neocortical neurons

Primary cultures of cortical neurons were prepared at embryonic gestation day 16–18 (E16–E18) (D'Orsi et al., 2016). To isolate cortical neurons, hysterectomies of the uterus of pregnant female mice were carried out after euthanizing mice by cervical dislocation. The cerebral cortices were isolated from each embryo and pooled in a dissection medium on ice [PBS with 0.25% glucose, 0.3% bovine serum albumin (BSA)]. Tissue was incubated with 0.25% trypsin-EDTA at 37°C for 15 min. After incubation, trypsinization was stopped by the addition of fresh plating medium (minimal essential medium containing 5% fetal bovine serum, 5% horse serum, 100°U/ml penicillin/streptomycin, 0.5 mM L-glutamine, and 0.6% D-glucose). Neurons were then dissociated by gentle pipetting, and after centrifugation (1,500 rpm, 3 min), medium containing trypsin was aspirated. Neocortical neurons were resuspended in plating medium, plated at 2 × 10^5^ cells per cm^2^ on poly-D-lysine-coated plates (final concentration of 5 μg/ml), and then incubated at 37°C and 5% CO_2_. The plating medium was exchanged with 50% feeding medium (neurobasal containing 100°U/ml of penicillin/streptomycin, 2% B27 and 0.5 mM L-glutamine) and 50% plating medium with additional mitotic inhibitor cytosine arabinofuranoside (600 nM). Two days later, the medium was again exchanged with complete feeding medium. All experiments were performed on day 8–11 *in vitro* (DIV).

### Preparation of mouse embryonic fibroblasts

Cultures of mouse embryonic fibroblasts were prepared at embryonic gestation day 12–14 as described by Durkin et al. (2013). Briefly, pregnant female mice were euthanized by cervical dislocation, and hysterectomy of the uterus was carried out. Embryos were taken out into a petri dish with PBS, and head, liver, and heart were removed. Embryos were subsequently moved into a dish with trypsin-EDTA solution and minced into 1–2 mm pieces. The resulting suspension was subjected to repeated pipetting- and incubation-steps (10 min at 37°C) and finally cultured in 20-ml medium (DMEM 4.5 g/L glucose, 10% fetal bovine serum, 2 mM L-glutamine, 100°U/ml penicillin/streptomycin). Cells of three embryos of the same genotype were combined in one T75 flask. Cells were passaged 1:2 when confluent up until passage 9.

### Generation of BOK-deficient cell lines

A MISSION^®^ shRNA vector for gene silencing of *BOK* (clone number TRCN0000033539) in mammalian cells and scrambled control containing plasmids were obtained from Sigma. Lentiviral particles were produced as previously described (Walter et al., 2015) by co-transfecting human HEK293TN cells with viral envelope protein encoding vector pMD2.G (Addgene, Cambridge, MA, United States) and packaging protein coding vector psPAX2 (Addgene) together with the MISSION^®^ shRNA containing pLKO.1-puro vector.

HEK293TN cells were cultivated in DMEM medium (4.5 g/L glucose), supplemented with 10% fetal calf serum, 2 mM L-glutamine, and 100°U/ml of penicillin/streptomycin. The supernatant containing the viral particles was harvested 48 and 72 h after transfection, centrifuged for 10 min at 3,000 ×*g* and stored at −80°C.

For viral transduction MEF cells, human SH-SY5Y cell neuroblastoma cells or SH-SY5Y-derived ER stress reporter cell lines grown in DMEM/Ham’s F12 (1:1 mixture) culture medium (Lonza) supplemented with 15% fetal calf serum, 2 mM L-glutamine, and 100°U/ml of penicillin/streptomycin were seeded at a density of 3 × 10^4^ cells/well in a 12-well plate and incubated with 250 µl virus suspension in 1 ml medium and 5 μg/ml polybrene per well. The cells were centrifuged at 1,000 ×*g* at 20°C for 90 min. The medium was changed after 24 h. The transduced cells were moved to Nunc®Micro Well optical bottom 96-well plates 72 h after transfection for time-lapse imaging. For selection of stable BOK-deficient cell lines, SH-SY5Y cells were treated with 5 μg/ml puromycin for 2 weeks. All cell lines were subjected to regular tests for mycoplasma using PCR Mycoplasma Test Kit 1/C (PromoCell GmbH, Heidelberg, Germany).

### Transduction of mouse embryonic fibroblast cells with PERK-reporter construct

The PERK-reporter plasmid was generated as described in Walter et al. (2015). The reporter region 5′UTR-ATF4-YFP was then sub-cloned into the viral expression vector pLVX using *Xho*I and *Xba*I restriction sites. Lentiviral particles containing pLVX-PERK-reporter or pLVX ctrl vector were generated as described previously.

### Transfection

For single-cell time-lapse experiments, MEF WT and *Bok* ^*−/−*^ cells were cultivated on cover glass bottom dishes (Willco Wells BV, Netherlands) at 5,000–10,000 cells per dish. For FRAP experiments, cells were transfected after 2–3 days in culture medium mixed with 10% OptiMEM containing 0.7 μl Lipofectamine 2000 and 0.5 μg of BiP-eGFP (Lai et al., 2010) plasmid for 4 h. Subsequently, cells were left for 48 h in culture medium before being subjected to FRAP imaging. For reconstitution of BOK function, BOK-kd or scram control IRE1-reporter cells were seeded in optical bottom 96-well plates at a density of 8,000 cells/well. After 24 h in culture, cells were transfected with the hBok (CDS)-pcDNA plasmid (a gift of Prof. Kaufmann, University of Bern) or pcDNA3.1 control plasmid using 25 ng DNA and 0.1 μl Metafectene (Biontex Laboratories GmbH, Muenchen, Germany) per well following the manufacturer’s instructions. For ER-calcium imaging experiments, cells were transfected after 2–3 days in culture medium mixed with 10% OptiMEM containing 0.7 μl Lipofectamine 2000 and 0.5 μg of ERD1cpv (Palmer et al., 2006) plasmid for 4 h. Subsequently, cells were left for 48 h in culture medium before being subjected to imaging of ER-calcium kinetics.

### Quantitative real-time PCR

Total RNA was extracted using the RNeasy minikit (Qiagen, Hilden Germany). First-strand cDNA synthesis was performed using 0.7 μg total RNA as template, reverse transcribed using Superscript III (Invitrogen) primed with 5 ng random hexamers following the manufacturer’s instructions. Quantitative real-time PCR (RT-qPCR) was performed using the TaqMan StepOne (Thermo Fisher) and QuantiTech SYBR green PCR kit (Qiagen) as per the manufacturer’s protocol. The following primers were used at a final concentration of 10 μM: *GRP78* for 5′-CTG​GCA​AGA​TGA​AGC​TCT​CC-3′, rev 5′-GGA​GTG​AAG​GCG​ACA​TAG​GA-3′’; *IRE1* for 5′- CTG​ACG​CCC​ACT​CTG​TAT​GTT-3′, rev 5′-CAA​TGG​TGA​CGC​CAT​CAG​TCT-3′; *BOK* for 5′- CGA​GAT​CAT​GGA​CGC​CTT​TGA-3′, rev 5′’-ATC​ATC​TCC​AGC​TCA​TCG​CC-3′; *ACTIN* for 5′-TCA​CCC​ACA​CTG​TGC​CCA​TCT​ACG​A-3′, rev 5′- CAG​CGG​AAC​CGC​TCA​TTG​CCA​ATG​G-3′. The PCRs were performed in 20-μl volumes with the following parameters: 95°C for 15 min, followed by 40 cycles of 94°C for 15 s, 58°C for 30 s, and 72°C for 30 s. The generation of specific PCR products was confirmed by melting curve analysis. The data were analyzed using the TaqMan StepOne software, with all samples normalized to β-actin.

### Immunocytochemistry

WT and *Bok*-deficient neurons grown on 13-mm coverslips were fixed with 4% paraformaldehyde for 15 min, permeabilized in PBS containing 0.1% Triton X-100, washed three times with PBS, and blocked for 1 h in 5% goat serum in PBS. Neurons were then incubated for 2 h with an anti-KDEL antibody diluted 1:100 (10C3, SPA827, Assay Designs) in 5% serum in PBS. Primary antibodies were detected using a 1:250 dilution of TRITC-conjugated goat anti-rabbit secondary antibody (Jackson ImmunoResearch, Plymouth, PA, United States) for 1 h. Coverslips were then transferred to glass slides with DAPI (4′,6-diamidino-2-phenylindole) mounting medium and sealed around the edges with clear varnish. Images of stained cells for quantification were captured with the LSM 7.10 confocal microscope equipped with a 63 × 1.4 NA oil immersion objective (Carl Zeiss). All microscope settings including laser intensity and scan time were kept constant for the whole set of experiments. All images were processed and analyzed using MetaMorph Software version 7.5 (Universal Imaging Co.), and the data presented as fluorescence intensity in arbitrary units (AU).

### Western blotting

Preparation of cell lysates from cortical neurons and mouse tissues and Western blotting were carried out as previously described (Reimertz et al., 2003). Lysates from SH-SY5Y were prepared, and gel electrophoresis performed as previously described (Walter et al., 2015). Proteins were transferred for 10 min at 1.3 A onto a nitrocellulose membrane using the Power Blotter XL and Power Blotter Select Transfer Stacks (Invitrogen, Carlsbad, CA, United States).

The resulting blots were probed with the following primary antibodies: mouse monoclonal anti-KDEL antibody (1:1,000) (10C3, SPA827, Assay Designs, Ann Arbor, MI, United States); rabbit polyclonal anti-IRE1α (3294; Cell Signaling Technology, Inc., Danvers, MA, United States); mouse monoclonal anti-XBP1 (1:500) (647501; BioLegend, San Diego, CA, United States); rabbit polyclonal anti-PERK (3192S; Cell Signaling); rabbit polyclonal anti-eIF2α (9722S; Cell Signaling); rabbit polyclonal anti-P-eIF2α (9721S; Cell Signaling); rabbit monoclonal BOK antibody (clone 1–5) (1:250) (Echeverry et al., 2013); mouse monoclonal anti-β-actin (1:10,000) (AC-15, A5441; Sigma); mouse monoclonal anti-tubulin antibody (1:1,000) (Sigma). Secondary antibodies conjugated to horseradish peroxidase (Thermo Fisher Scientific, Dublin, Ireland) were detected using Immobilon chemiluminescent HRP substrate (Millipore, Cork, Ireland). Blots were imaged using LAS-4000 imaging system (Fujifilm, Sheffield, United Kingdom).

### High-content time-lapse imaging

BOK-kd SH-SY5Y cells and control cells or BOK-kd ER stress reporter cell lines and respective control cells were seeded on Nunc^®^Micro Well optical bottom 96-well plates and incubated overnight. Four hours prior to imaging, the cells were stained with 100 ng/ml Hoechst 33588 (Invitrogen, Biosciences) and 2 μg/ml propidium iodide (PI) (Sigma, Ireland). Immediately before imaging, the cells were treated with thapsigargin, tunicamycin, brefeldin A, or the same volume of DMSO (five wells per treatment). The plate was mounted on the stage incubator (37°C, 5% CO_2_) of a high-content screening system (ArrayScan VTI, Cellomics, United States), equipped with a ×10 PlanApo objective lens (NA 0.45) and a monochrome CCD camera (Orca AG, Hamamatsu, Japan), modified with an LED-based light source (Sola, AHF, Germany). The following filter sets were used: Hoechst excitation 387 ± 11 nm, quad band emission filter 440/521/607/700; PI excitation 504 ± 12 nm, emission 645 ± 75 nm; YFP excitation 504 ± 12 nm, emission 542 ± 27 nm (Chroma and Semrock, AHF, Germany). Images were taken of nine fields of view per well in 1-h intervals for 48 h, with 1024 × 1024 pixel resolution (6.45 μm pixel size). Only for the first set of images, the image-based autofocus was active. Imaging data were analyzed as described before (Walter et al., 2018); briefly: Cell Profiler software (cell profiler version 2.2, the Broad Institute, United States) (Kamentsky et al., 2011) was used in conjunction with a purpose built MATLAB script (MATLAB; MathWorks Inc., Cambridge, United Kingdom) to identify objects, ascertain their mean fluorescence intensity, and plot the results.

### Live-cell imaging and measurement of intracellular kinetics of Ca^2+^

SHSY5Y cells stably expressing shRNA against *BOK* or scrambled shRNA were grown on cover glass bottom dishes (WillCo Wells BV, Netherlands) for 24 h before they were stained with 5 μM Fluo-4-AM (BioSciences, Ireland) for 45 min in the incubator. The medium was then replaced with Krebs buffer with 1.2 mM CaCl_2_ or100 μM EGTA and covered with embryo-tested mineral oil (Sigma, Ireland) to prevent evaporation. The cells were then placed on the heated stage of an LSM 5 LIVE equipped with a 40 × 1.3 NA oil immersion objective run by ZEN software (Carl Zeiss, United Kingdom) and imaged using the 488 nm excitation at 2%, every 10 s. After a 5-min baseline, 3 μM thapsigargin was added to the cells, and they were imaged for another 25 min. The average intracellular fluorescence intensity of Fluo-4 was analyzed using ImageJ (version 1.52p-r, Wayne Rasband, NIH, United States). Areas under the curve were analyzed using GraphPad Prism 5, Kruskal–Wallis, and Dunn’s tests.

### Fluorescence recovery after photobleaching

Cover glass bottom dishes with transfected cells in culture medium, which was then covered by embryo-tested mineral oil (Sigma, Ireland) to prevent evaporation, were mounted on the heated stage of a confocal microscope (LSM 710, Carl Zeiss, United Kingdom) and incubated in 5% CO_2_. A region of interest of 512 × 256 pixels containing the cell of interest was selected and then a spot with a diameter of 1.7 μm in the upper half of the image was selected as bleach spot. Images were acquired at 2 Hz. For the bleaching, 100% of the 488-nm line of the argon laser was used with 20 sweeps at ¼ of the imaging scan speed to sufficiently bleach the spot. Three to five spots were selected in the ER area expressing BIP-eGFP of each cell. During the 30 s experiments, the focus was kept stable with a hardware-based focus stabilizer (Definite Focus v1, Carl Zeiss, United Kingdom). After the initial experiment, cells were treated with 3 μM tunicamycin and kept in the incubator. FRAP experiments were repeated 6 h after treatment.

### Fluorescence recovery after photobleaching analysis

LSM images were processed and analyzed using FiJi/ImageJ (version 1.52p-r). The mean intensity of a circular area covering the bleach spot exactly and of a reference area, which was only scanned, not bleached, were analyzed using the multi-measure option of the ROI manager tool. Results were saved in csv format and imported into the FRAP analyzer tool (FRAP Analyzer, v 1.0.5, Halavatyi A., Cytoskeleton and Cell Plasticity Laboratory, University of Luxembourg; based on Sprague et al. (2004) Equation 6), normalized using double normalization and then fitted using the fit function for binding and diffusion in a circular spot. The four fit parameters R (depicting the recovery), k_on_ (binding constant), k_off_ (dissociation constant), and D (diffusion coefficient) were then compared for the different conditions (WT vs. *Bok* ^−/−^ 0 and 6 h after 3 μm tunicamycin) using GraphPad Prism 5, Kruskal–Wallis and Dunn’s tests.

### Statistical analysis

Data are given as means ± SEM (standard errors of the means). Data were analyzed using one-way analysis of variance (ANOVA) followed by Tukey’s post-hoc test or Student’s t-test for two-group comparison. *p*-values < 0.05 were considered to be statistically significant.

## Data Availability

The raw data supporting the conclusion of this article will be made available by the authors, without undue reservation.

## Abbreviations

ANOVA, analysis of variance; BFA, brefeldin A; BH, Bcl-2 homology domain; Bok, Bcl-2-related ovarian killer; DIV, day in vitro; ER, endoplasmic reticulum; FRAP, fluorescence recovery after photobleaching; HCS, high-content screening; MOMP, mitochondrial outer membrane potential; Tg, thapsigargin; Tm, tunicamycin; UPR, unfolded protein response; WT, wild type.
