# Supplementary material for: BOK controls ER proteostasis and physiological ER stress responses in neurons
Source: Front Cell Dev Biol. 2022 Aug 15;10:915065. doi: 10.3389/fcell.2022.915065 (PMC9434404; doi:10.3389/fcell.2022.915065)
Supplement: Supplementary file 1 [file DataSheet1.docx]

Supplementary Material

# Supplementary Figures

Supplementary Figure 1


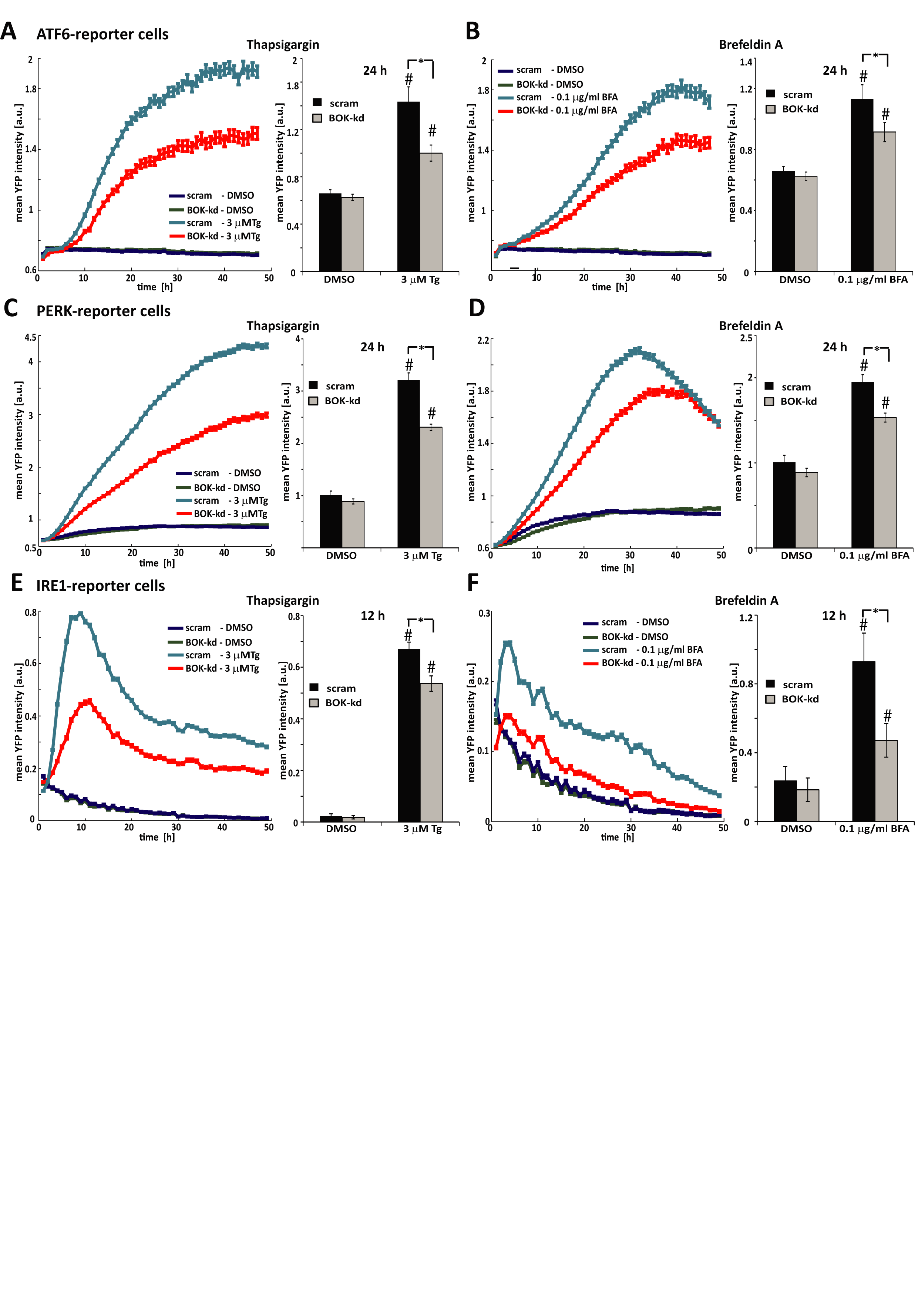


**Supplementary Figure 1.** SH-SY5Y cell lines stably expressing the ATF6-, PERK- or IRE1-reporter were transduced with shRNA against BOK or scrambled control vector. 72 h after transductions cells were stained with Hoechst and PI and exposed to ER-stress. Images were taken at 1 h intervals starting immediately after treatment for 48 h using high content time lapse live cell imaging. A) Mean YFP intensity over time of ATF6- reporter cells silenced for BOK-expression and control in response to 3 mM Tg or 0.1 % DMSO or B) 0.1 mg/ml BFA or 0.1 % DMSO. Error bars indicate SEM of n=5 wells. Bar graphs show mean YFP intensity 24 h after treatment. Error bars indicate SEM of n=15 wells of 3 independent experiments. T-tests were performed comparing BOK-kd and scrambled control groups. * indicates p<0.01 C) Mean YFP intensity over time of PERK- reporter cells silenced for BOK-expression and control in response to 3 mM Tg or 0.1 % DMSO or D) 0.1 mg/ml BFA or 0.1 % DMSO. Error bars indicate SEM of n=5 wells. Bar graphs show mean YFP intensity 24 h after treatment. Error bars indicate SEM of n=15 wells of 3 independent experiments. T-tests were performed comparing BOK-kd and scrambled control groups. * indicates p<0.01 E) Mean YFP intensity over time of IRE1- reporter cells silenced for BOK-expression and control in response to 3 mM Tg or 0.1 % DMSO or F) 0.1 mg/ml BFA or 0.1 % DMSO. Error bars indicate SEM of n=5 wells. Bar graphs show mean YFP intensity 12 h after treatment. Error bars indicate SEM of n=15 wells of 3 independent experiments. T-tests were performed comparing BOK-kd and scrambled control groups (* indicates p<0.01) or DMSO- and Tunicamycin treated groups (# indicates p<0.01)

Supplementary Figure 2


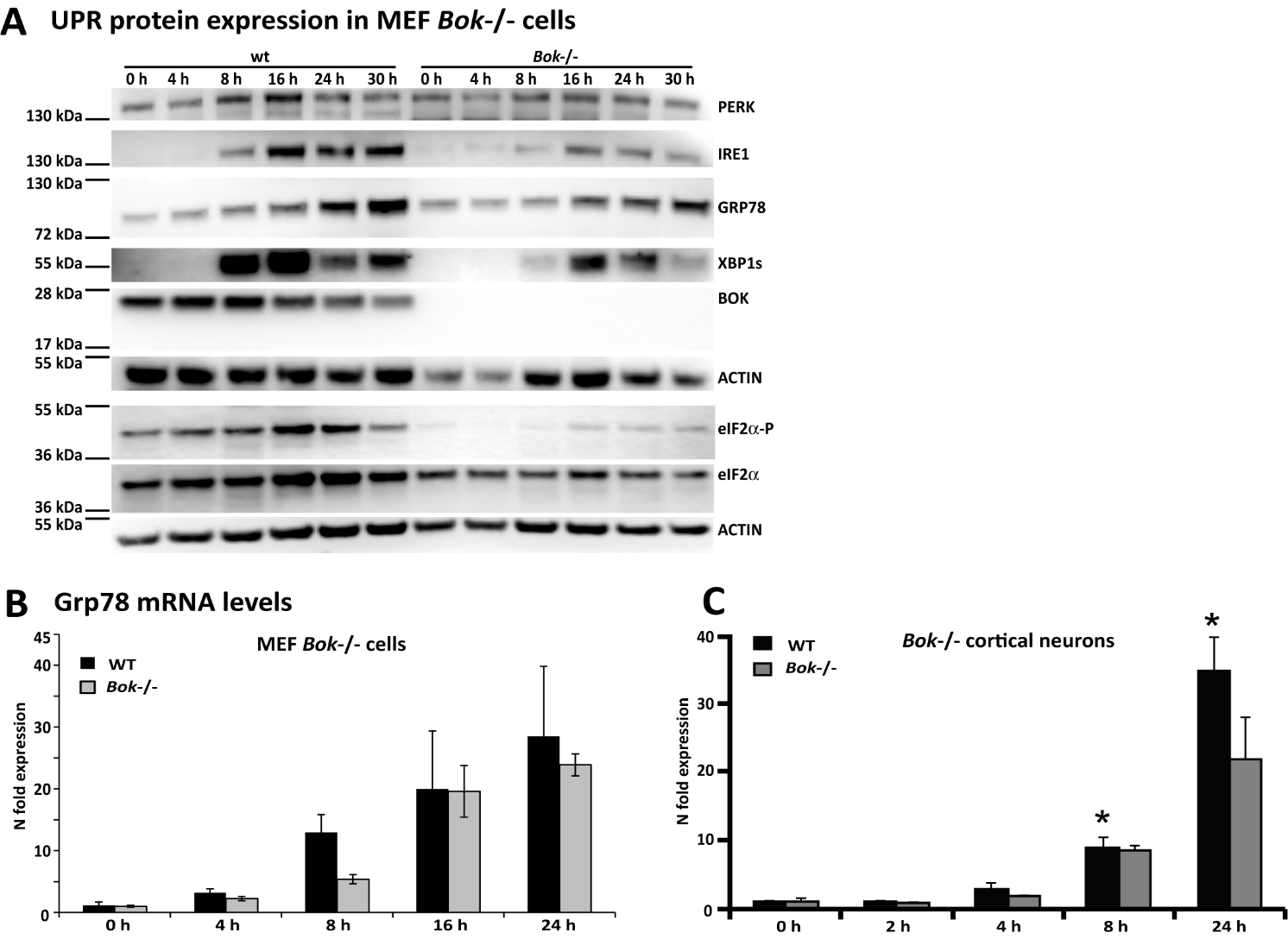


**Supplementary Figure 2**: Mouse embryonic fibroblasts from WT or *Bok*-/- mice were treated with 3 μM Tm and harvested at times indicated. **A)** Expression of UPR proteins was analysed by Western Blotting using antibodies against PERK, IRE1, KDEL, spliced XBP1s, EIF2α-P, EIF2α and BOK. Actin served as loading control. The experiment was repeated with similar results.

**B)** Real time qPCR analysis of *Grp78* mRNA levels in WT and *Bok*-/- MEF cells treated with 3 μM Tm Results were normalized to β-actin levels and expressed relative to 0 h WT or *Bok*-/- cells, respectively (mean of n=3 wells , error bars indicate SEM, *.* T-tests were performed comparing wt and *Bok*-/- * indicates p<0.01 **C)** Real-time qPCR of *Grp78* mRNA levels in cortical neurons from WT and *Bok^-/-^* mice treated with 3 μM Tm for the indicated time periods. The relative mRNA expression levels were assessed by RT-qPCR and normalized to β-actin mRNA levels. Expression levels were normalized to control-treated cells and data are represented as means ± SEM from three wells. Experiments were repeated at least three times with different preparations and similar results. *indicates statistical significance (*p* ≤ 0.05).

Supplementary Figure 3


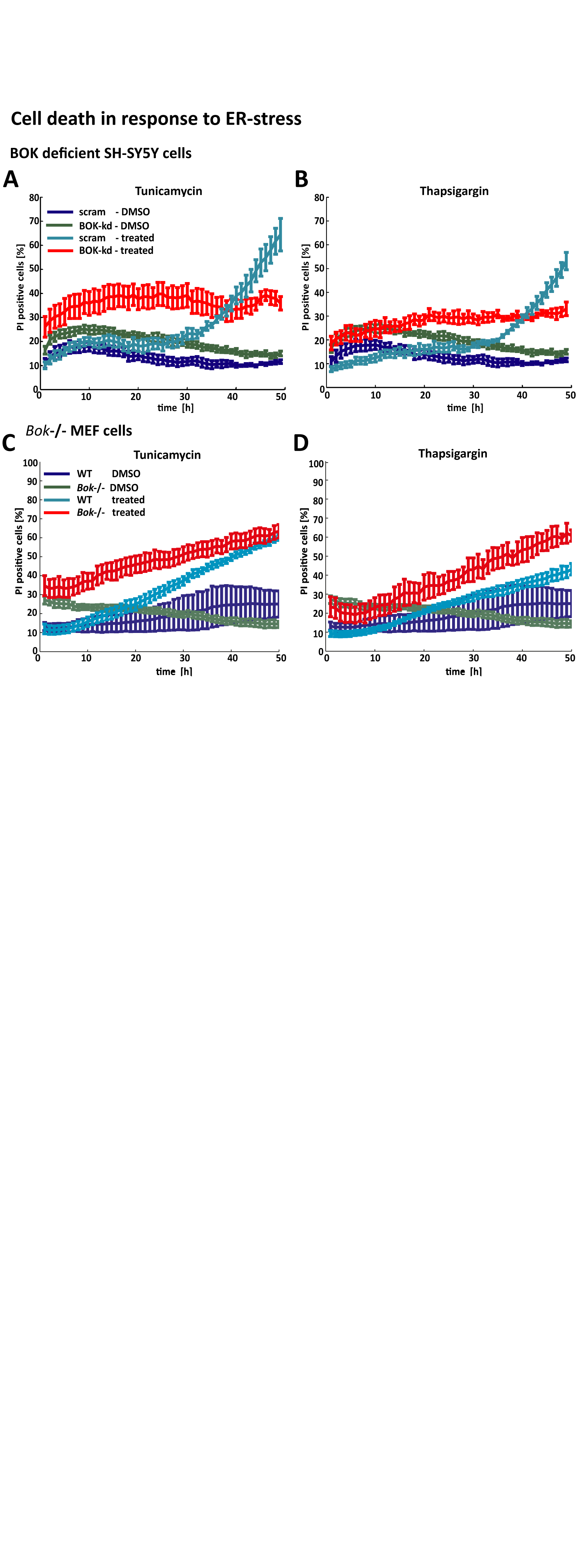


**Supplementary Figure 3:** To investigate cell death cells were stained with Hoechst and PI and treated as indicated. Images were taken at 1 h intervals starting immediately after treatment for 48 h using high content time lapse live cell imaging. **A-B)** SH-SY5Y cells stably expressing shRNA against *BOK* or control vector were treated with **A)** 3 μM Tm or **B)** 3 μM Tg. Percentage of PI positive cells over time in response to treatment as indicated or 0.1 % DMSO ctrl was plotted. Error bars indicate SEM of n=5 wells of 1 representative experiment. **C-D)** *Bok***-/-** or WT MEF cells were treated with **C)** 3 μM Tm or **D)** 3 μM Tg. Percentage of PI positive cells over time in response to treatment as indicated or 0.1 % DMSO ctrl was plotted. Error bars indicate SEM of n=5 wells of 1 representative experiment.
